# Editorial: Imaging to guide treatment in brain tumors

**DOI:** 10.3389/fonc.2026.2012733

**Published:** 2026-10-14

**Authors:** Anouk van der Hoorn, Stefanie Thust, Jan Petr, Vera C. Keil

**Affiliations:** 1 Department of Radiology, University Medical Center Groningen, University of Groningen, Groningen, Netherlands; 2 Precision Imaging, School of Medicine, University of Nottingham, Nottingham, United Kingdom; 3 Nottingham NIHR Biomedical Research Centre, Nottingham, United Kingdom; 4 Helmholtz-Zentrum Dresden Rossendorf, Institute of Radiopharmaceutical Cancer Research, Dresden, Germany; 5 Amsterdam UMC, Dept of Radiology and Nuclear Medicine, Amsterdam, Netherlands; 6 Cancer Center Amsterdam, Imaging and Biomarkers, Amsterdam, Netherlands

**Keywords:** brain tumour, cancer, glioma, imaging, lymphoma, neuro-oncology

Brain tumors are highly heterogeneous, resulting in various malignancy grades, but also a high variability in imaging phenotypes. From diagnosis to long-term follow-up, clinicians need imaging to provide more than a lesion’s size and location. They need to understand the characteristics of the tumor, how it will behave, whether a treatment is effective, and how that treatment affects the healthy brain around the tumor. Magnetic resonance imaging (MRI) and positron emission tomography (PET) have the potential to address these questions early, non-invasively, and serially, whereby both have grown in sophistication and diversity. Our Research Topic “Imaging to Guide Treatment in Brain Tumors” set out to survey that progress in adults with primary and metastatic brain tumors, covering all aspects of treatment-related imaging. Reflecting the complexity of brain tumor imaging, the thirteen articles in this collection span gliomas, primary central nervous system lymphoma (PCNSL), meningiomas, and brain metastases, and range from quantitative physiological imaging to deep learning at different time points during the treatment pathway.

A first hot topic is the shift from depicting anatomy to measuring physiology. Petr et al. compared two MRI perfusion techniques, arterial spin labeling (ASL) and dynamic susceptibility contrast (DSC), with ^15^O-H_2_O PET, the reference standard for perfusion measurement, in a group of patients with diffuse glioma. ASL showed comparable agreement with PET in normal and tumor tissue, supporting its use as a non-invasive alternative to DSC. However, both methods bear individual challenges, and therefore they appear complementary rather than interchangeable, and combining them deserves further study. Diffusion imaging offers a similar window on tumor biology: Zhu et al. showed in adult-type diffuse gliomas that the relative minimum apparent diffusion coefficient (ADC) was higher in MGMT-methylated tumors and discriminated methylation status in IDH-wildtype gliomas, while ADC parameters correlated inversely with the Ki-67 proliferation index, underscoring that ADC also carries information on glioma proliferation. Defining MGMT status is clinically relevant, as methylated tumours are more sensitive to alkylating chemotherapy (Temozolomide) with prognostic implications.

Another topical ambition is to extract prognostically impactful data, like molecular signatures, from routine imaging. Christodoulou et al. developed an explainable three-dimensional convolutional neural network ensemble to predict IDH and MGMT status, trained on multi-institutional cohorts and tested this on an independent external cohort. IDH prediction performed strongly in testing and held up moderately in external validation, with FLAIR imaging contributing most to the model’s decisions. This may hint at another topic: the future relevance of post-gadolinium sequences in brain tumor imaging. MGMT prediction, by contrast, generalized poorly despite promising internal results. A companion study from the same group (Christodoulou et al.) used a modality-adaptive radiomics framework for predicting methylation status in patients with high-grade glioma. The model integrates DWI and ASL data and adjusts automatically when they are unavailable, an everyday reality in heterogeneous clinical settings. While it showed modest performance and advanced MRI importance, the added value of advanced MRI still remains to be shown in a large cross-center study. Astaraki et al. added spatial information by quantifying tumor distribution across brain anatomical structures in a large sample drawn from several independent datasets, illustrating the importance of cross-center data availability for progress in our field. Again, IDH status was predicted well within datasets but less so across them, whereas MGMT status and overall survival remained difficult to predict from images alone. The message of these three articles is consistent: IDH genotyping is within reach of imaging, likely even with simpler visual tools such as the T2-FLAIR-mismatch, while MGMT methylation is evasive for imaging biomarkers. The importance of external validation, rational explainability, and clinical-utility testing is essential before such tools can guide treatment. Hu et al. extend the discussion to brain metastases in a mini review of artificial intelligence for lesion detection and segmentation, differential diagnosis, identification of the primary tumor, and discrimination of radiation necrosis from recurrence, tracing progress from early convolutional networks to adaptive frameworks such as nnU-Net, which detect and delineate lesions with high sensitivity.

The spectrum of brain tumor imaging research does not stop with metastases and gliomas. Two articles address PCNSL, where response is traditionally judged on contrast enhancement. Hou and Liu identified that non-enhancing T2-FLAIR hyperintense lesions remote from the enhancing tumor occurred in a subset that regressed or resolved with chemotherapy. These lesions were associated with multiple and bilateral enhancing lesions. Further studies will investigate if an explanation is early neoplastic infiltration ahead of blood–brain barrier disruption. This again suggests an important role for contrast-free sequences in disease monitoring and early biomarker development possibly to be considered for future imaging guidelines. Reporting negative findings is essential in imaging biomarker research: Menon et al. report that in patients when uniformly treated with MATRix chemotherapy, baseline contrast-enhancing tumor volume and ADC metrics did not predict overall or progression-free survival, underlining that standard MRI measures alone are insufficient for prognostication and that integration with molecular and biological data is needed. In meningioma, Spolaore et al. transferred voxel-based lesion mapping, a technique from cognitive neuroscience, to neurosurgical oncology showing the relevance of crosspollination in techniques. In a large surgical series they related tumor location and the dural attachment zone to histology, symptoms, resection completeness and postoperative deficits. Most importantly, incomplete resection was more frequent in medial sphenoid wing meningiomas, information that can inform preoperative counselling and surgical planning.

Several publications for this Research Topic report on the assessment of treatment response and treatment-related injury. Zarębska et al. review the role of FET-PET in recurrent glioblastoma from a radiation oncologist’s perspective. Standard MRI struggles to separate treatment related imaging abnormalities from tumor progression, whereas amino-acid PET can support reirradiation from patient selection to response assessment. Injury to the healthy brain is addressed by Kłos et al., who studied diffusion tensor imaging in patients with lower-grade gliomas. Fractional anisotropy (FA) in contralateral white matter decreased significantly at the highest radiotherapy doses, whereas grey matter FA and ADC were unchanged, and the decrease was unrelated to the volume of FLAIR hyperintensity, indicating that DTI and FLAIR capture different aspects of radiation-induced brain damage. Two further articles pair imaging with unconventional markers. In the prospective GMaPIC trial, Casile et al. evaluated a non-invasive intracranial pressure estimate based on otoacoustic emissions (OAE) in patients with high-grade glioma undergoing radiotherapy. OAE are faint sounds emitted by outer hair cells of the normal inner ear, which may become altered in disease; their variation was significantly associated with changes in T2 FLAIR edema volume on MRI – a simple bedside measurement may one day complement imaging-based monitoring. Finally, Rafaelian et al. used routine follow-up MRI to track the velocity of temporalis muscle wasting in patients operated on for cerebral metastasis. Slower temporalis wasting was associated with longer survival, both overall and after surgery, turning scans acquired for tumor surveillance into a dynamic marker of patient frailty.

These articles illustrate three shifts. Imaging is increasingly quantitative, whether in perfusion measured against a physical reference, diffusion metrics, dose–response relations in normal-appearing white matter, or the rate of muscle loss. Brain tumor imaging is becoming molecular and computational, benefitting from machine learning that is increasingly explainable and externally tested – but also still in need of clinical availability and validation. And research is becoming integrative, by combining MRI with PET, by non-imaging monitoring, and through inclusion of clinical and molecular data. Equally important are the negative results: modest external performance for MGMT prediction, no prognostic value of baseline MRI in MATRix-treated lymphoma, and a frequent struggle with small data samples. Collaborative imaging research is needed more than ever in this field. Societies and working groups must stay active.

The collection also leaves gaps. Treatment-related imaging abnormalities were discussed in reviews but not examined in dedicated original studies. The cognitive consequences of radiotherapy, imaging changes after immunotherapy, and optimal follow-up protocols remain largely open, illustrating that multidisciplinary research is scarce. Priorities for future work include harmonized acquisition protocols to be used in multicenter prospective validation to assess treatment outcomes that incorporate patient-relevant outcomes. We thank all authors, reviewers and the Frontiers editorial staff for making this Research Topic possible.

